# Sepsis-induced cardiac dysfunction: mitochondria and energy metabolism

**DOI:** 10.1186/s40635-025-00728-w

**Published:** 2025-02-18

**Authors:** Xueting Yu, Jie Gao, Chunxiang Zhang

**Affiliations:** 1 Key Laboratory of Medical Electrophysiology, Ministry of Education, Institute of Cardiovascular Research, the Affiliated Hospital of Southwest Medical University, Southwest Medical University, Luzhou, Sichuan China; 2 Department of Cardiology, Institute of Cardiovascular Research, the Affiliated Hospital of Southwest Medical University, Southwest Medical University, Luzhou, Sichuan China; 3 FACC, Southwest Medical University, Luzhou, Sichuan China

**Keywords:** Sepsis, Cardiac dysfunction, Mitochondria, Energy metabolism

## Abstract

Sepsis is a life-threatening multi-organ dysfunction syndrome caused by dysregulated host response to infection, posing a significant global healthcare challenge. Sepsis-induced myocardial dysfunction (SIMD) is a common complication of sepsis, significantly increasing mortality due to its high energy demands and low compensatory reserves. The substantial mitochondrial damage rather than cell apoptosis in SIMD suggests disrupted cardiac energy metabolism as a crucial pathophysiological mechanism. Therefore, we systematically reviewed the mechanisms underlying energy metabolism dysfunction in SIMD, including alterations in myocardial cell energy metabolism substrates, excitation–contraction coupling processes, mitochondrial dysfunction, and mitochondrial autophagy and biogenesis, summarizing potential therapeutic targets within them.

## Introduction

According to the 2021 edition of the International Guidelines for the Diagnosis and Treatment of Sepsis, sepsis is defined as a life-threatening multi-organ dysfunction syndrome caused by dysregulated host response to infection, commonly encountered in intensive care units as a deadly disease. Sepsis manifests across all age groups clinically, with higher mortality rates observed among infants and the elderly[1]. Research indicates that over 30 million people worldwide suffer from sepsis annually, with an average mortality rate of 24.39% within 30 days, and a 30-day mortality rate of 34.7% among septic shock patients, making sepsis become a significant global healthcare issue[2, 3].

Sepsis can lead to the development of multi-organ dysfunction due to inflammation, oxidative stress, and coagulation abnormalities. Sepsis-induced myocardial dysfunction (SIMD) is one of the most common complications of sepsis, with a prevalence of approximately 10–70% in septic patients, and even leads to an increased mortality rate of 70–90% in septic patients[3]. SIMD currently has no clear clinical diagnostic criteria, and in its early stages, it usually manifests as an overall systolic or diastolic dysfunction of the heart, with a common clinical manifestation of reduced left ventricular (LV) ejection fraction, of which approximately 40% of patients will present with acute heart failure, and the main biomarkers are serum troponin, hormones B-type natriuretic peptide (BNP) and N-terminal pro-BNP (NT-proBNP) [4, 5].

Clinical treatments are similar to those for sepsis and mainly include: (1) etiologic treatment, early control of the source of infection and use of effective anti-microbial drugs; (2) supportive treatment, including fluid resuscitation, vasoactive drugs, and organ supportive treatments such as mechanical ventilation, renal replacement therapy, and ECMO according to the severity of organ dysfunction [6]. Patients with sepsis often require surgery for removal of the source of infection, according to the latest expert consensus on perioperative management of patients with sepsis, preoperative comprehensive and integrated assessment of the past medical history of patients with sepsis, organ damage, as well as combined with the characteristics of the surgery itself is required, intraoperative in addition to routine monitoring, should increase the relevant organ function, tissue perfusion and thromboelastography (TEG), postoperative attention to immune conditioning, and for the patients who have been transferred to the ICU, a restrictive fluid resuscitation program should be adopted, and attention should be paid to the protection of cardiac, pulmonary, renal and other organ functions [1, 7]. Nevertheless, the overall number of deaths among SIMD patients continues to rise.

Recent studies have shown that this unique cardiac dysfunction involves a significant disturbance in energy metabolism. Normal diastolic and systolic function of the heart is highly dependent on adenosine triphosphate (ATP), 95% of which is produced by oxidative phosphorylation of cardiomyocyte mitochondria. Studies have shown significant mitochondrial dysfunction in SIMD, resulting in an inability to supply sufficient ATP to maintain normal cardiomyocyte function[8].

Therefore, we believe that cardiomyocyte energy metabolism in SIMD is an area worthy of future investigation. In this article, we will review the mechanisms of changes in myocardial energy metabolism and abnormal mitochondrial function in SIMD, and explore potential therapeutic targets.

## Cardiac energy metabolism in SIMD

Adenosine triphosphate (ATP) is the sole direct source of energy for cardiac contraction and relaxation, but the storage capacity of ATP in cardiomyocytes is low. Therefore, the heart heavily relies on continuous ATP generation by mitochondria to maintain energy supply. Physiologically, The energy substrate of the heart is dominated by fatty acids (70%), supplemented by pyruvate (derived from glucose and lactate), ketone bodies and amino acids [9, 10].

In contrast, in the pathological state of SIMD, patients experience significant alterations in myocardial substrate uptake, impaired fatty acid oxidation, accumulation of large amounts of intermediary metabolites and mitochondrial dysfunction, leading to a pathological reduction in ATP production[10]. Unlike other organs, in SIMD patients, insulin resistance and inhibition of glucose utilization are common issues, meaning the reduced fatty acid oxidation in the heart cannot be compensated for by increased glucose metabolism[11]. At the same time, the heart obtains large amounts of lactate through aerobic glycolysis production and peripheral blood uptake, and uses it as a major energy substrate. This suggests that cardiomyocytes attempt to improve myocardial energy metabolism by adjusting energy substrates during SIMD, suggesting the importance of myocardial energy metabolism in SICM.

## Mitochondria: the metabolic hub

The normal physiological function of mitochondria depends on key proteins involved in electron transfer, the tricarboxylic acid (TCA) cycle, and the maintenance of mitochondrial structure, requiring coordinated action between nuclear DNA and mtDNA. Besides playing a critical role in energy metabolism, mitochondria also play important roles in cellular calcium homeostasis regulation, reactive oxygen and nitrogen species generation, and cell signaling[11, 12].

Sepsis is mainly due to bacterial endotoxin triggering pathogen-associated molecular patterns (PAMPs), damage-associated molecular patterns (DAMPs) and metabolism-associated molecular patterns (MAMPs), all of which appear to involve mitochondria as bridges[13]. Notably, in heart samples from patients with clinical SIMD, there was no detectable extensive myocardial ischemia and hypoxia, necrosis of cardiomyocytes or structural destruction of the myocardium, but ATP production, oxygen consumption, and glutathione levels were reduced in cardiac tissues; at the same time, significant mitochondrial swelling and rupture, disordered arrangement, reduced number, reduced mitochondrial enzyme activity, and accumulation of mitochondrial proteins in the cytoplasm were observed, in addition, mitochondrial DNA was also more susceptible to the effects and damage of LPS than nuclear DNA[14]. SIMD cardiomyocytes and cardiac immune cells exhibit significant metabolic reprogramming due to the level of glycolysis and the state of the mitochondria, which affect the intracellular environment and gene expression, and induce epigenetic changes by affecting the post-translational modifications of histones[15, 16].

**Fig. 1:**
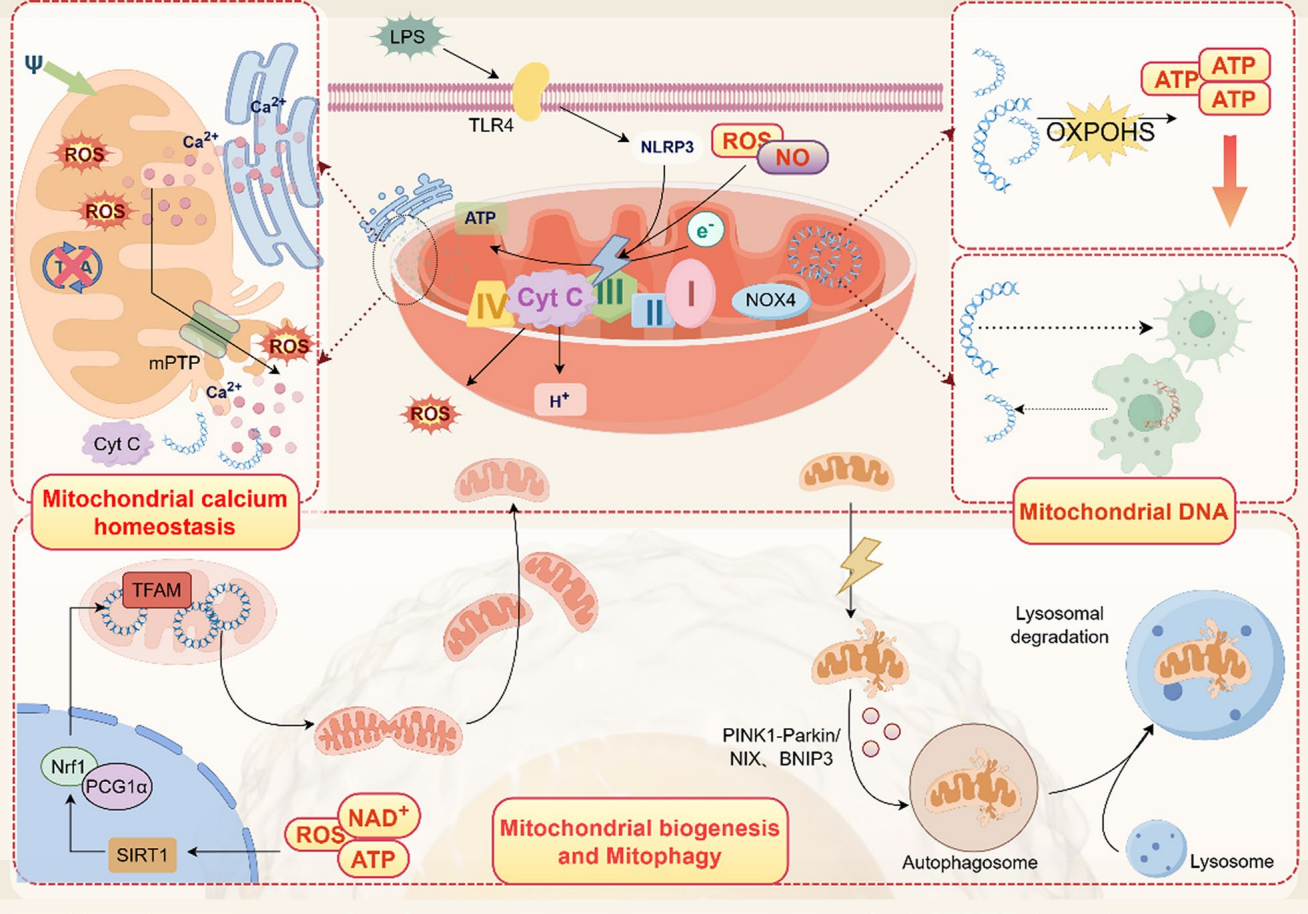
The pivotal role of mitochondria in the energy metabolism of systemic inflammatory response syndrome (SICM) is elucidated (by Figdraw). Stimulation by lipopolysaccharide (LPS) induces an elevation in reactive oxygen species (ROS) and nitric oxide (NO) levels within cells, leading to disruption of the mitochondrial respiratory chain. On one hand, this results in an increase in mitochondrial ROS levels, damage to mtDNA, disturbance of calcium homeostasis, rupture of mitochondria, and release of their contents. On the other hand, alterations in the intracellular milieu activate both mitochondrial autophagy and biogenesis

These results indicate that: 1. the cardiac dysfunction induced by sepsis is primarily a cellular dysfunction rather than a tissue pathological phenomenon; 2. mitochondria are both the primary victims and amplifiers of cellular dysfunction in SIMD. Therefore, it is necessary to explore the mechanisms of mitochondrial energy metabolism disorders in SIMD (Fig. 1).

### Oxidative stress: ROS and NO

Oxidative stress (OS) refers to an imbalance between cellular free radical production and antioxidant system, which produces ROS and reactive nitrosylated substances (RNS, mainly focusing on the impact of NO in SIMD) in excess of the cellular load thereby triggering a series of cascading signaling responses. In SIMD, excess ROS cause cytotoxicity, such as lipid peroxidation and nitrosylation, resulting in mitochondrial damage, which causes a collapse in energy metabolism and cardiomyocyte death. In contrast, nitric oxide (NO), in addition to directly affecting cardiomyocytes, induces vasodilation in a dose-dependent manner, decreasing vascular tone throughout the body and aggravating cardiac burden[17].

#### Regulation targets of ROS in SIMD

The main sources of ROS in SIMD are NADPH oxidases (NOXs) and the mitochondrial respiratory chain. NOXs are widely distributed transmembrane proteins that are the main source of ROS in the cytoplasm. Four of these isoforms, NOX1, NOX2, NOX4 and NOX5, were confirmed to be expressed in the heart[18]. High expression of NOX1 and NOX2 can cause downregulation of the transcriptional activity of PPARα/PCG1α in cardiomyocytes, inhibiting glucose and fatty acid metabolism. In vitro experiments have confirmed that inhibiting NOX2 can help maintain cellular calcium homeostasis and mitochondrial function, and relieve cardiomyocyte contractile dysfunction[19]. On the contrary, NOX4 helps trigger a series of adaptive stress responses in cardiomyocytes, such as inducing high expression of HIF1α, ATF4 and NRF2, increasing the synthesis of intermediates of the pentose phosphate pathway (PPP) and glutathione, inducing mitochondrial autophagy, and avoiding mitochondrial calcium overload[20]. NOX5 requires Ca^2+^ for its activation, and we speculate that its function may be related to intracellular calcium homeostasis and may affect the excitation–contraction coupling of cardiomyocytes[21]. It is worth noting that NOX5 is not expressed in rodents, which limits the current research. Therefore, follow-up studies need to consider other animal models.

Approximately 11 sites for ROS production exist on the mitochondrial respiratory complex in physiological states. These ROS are maintained at low physiological levels by a variety of antioxidant enzymes[22]. Recently, a novel mode of non-caspase-dependent programmed cell death—parthanatos—has attracted the attention of researchers, which is associated with the activation of PARP and structural damage to mitochondria. A study has shown that erlotinib can attenuate ROS-induced macrophage parthanatos by inhibiting the expression of TLR4 to improve survival in septic mice, and PARP inhibitors have been demonstrated to improve survival and reduce the incidence of organ failure in mice in animal experiments [23]. Currently, the FDA has approved the reintroduction of PARP inhibitors for the treatment of sepsis, while research on mitochondria-targeted antioxidants is actively advancing and has made some progress in animal experiments[24].

#### Regulation targets of NO in SIMD

In SIMD, inducible nitric oxide synthase (iNOS) is the major enzyme responsible for generating NO. Pathological sections of the hearts of a large number of animal and human patients have demonstrated that iNOS is in a state of overactivation in SICM. iNOS is in a state of overactivation, producing high concentrations of NO in the microgram range, and once activated, it can be expressed in various cells throughout the body until iNOS is degraded[25].

Mechanistically, NO can exacerbate mitochondrial damage through various pathways: 1. inhibiting the original activity of proteins containing heme–iron or iron–sulfur centers, such as inhibiting pyruvate dehydrogenase (PDC) and aconitase activity, and bind to the iron–copper center of cytochrome C (respiratory chain complex IV) to inhibit mitochondrial respiration[26]; 2. Nitrosation or nitration of proteins; 3. binding with generated O^2−^ to produce ONOO^−^, directly damaging proteins or DNA; 4. Activation of the cyclic guanosine monophosphate (cGMP) signaling pathway, which causes vasodilation and a negative inotropic effect on the heart muscle; 5. high concentrations of NO cause ribotoxicity, which leads to protein translation being blocked and triggers cell dysfunction[27]. Therefore, timely inactivation of iNOS or control of the release flow of NO is crucial in the treatment of SIMD.

Merck, AstraZeneca, and Pfizer have invested heavily in research on specific inhibitors designed to target the structure and binding mode of iNOS. However, the iNOS system involves a complex signal transduction system, and the clinical therapeutic effect in sepsis has not met expectations[28]. Non-selective iNOS inhibitors even increased the incidence of SIMD[29]. Recently, advancements have been made in the field of research, with the cGMP signaling pathway being a notable area of focus. The phosphodiesterase protein family (PDEs), acting as the second messenger of cGMP, represents an optimal target for the development of positive inotropic drugs and vasopressors. PDE inhibitors such as levosimendan and milrinone are currently undergoing phase 4 clinical trials and have demonstrated efficacy in enhancing cardiac contractility in patients with SIMD[30]. Additionally, these agents can be utilized to maintain blood pressure following catecholamine tolerance[31].

### Mitochondrial DNA metabolism in SIMD

A vicious cycle of metabolic disorders and tissue damage is observed in SIMD. Mitochondrial DNA (mtDNA) has been identified as a signaling molecule for metabolism and inflammation in organelles and intercellular connections. Mitochondrial DNA (mtDNA) exhibits two distinctive characteristics. Firstly, it is more susceptible to damage and lacks a self-repair mechanism. Secondly, it is readily identified by pattern recognition receptors (PRRs) as a foreign entity, triggering inflammatory signaling cascades and elevating the release of pro-inflammatory mediators[32, 33].

Accordingly, the detrimental effects of mtDNA can be mitigated through the implementation of the following strategies (Table 1):

1. The maintenance of mitochondrial stability to reduce the release of mtDNA;
2. The prompt removal of released mtDNA;
3. The inhibition of inflammatory signals. On the one hand, a review published by Central South University in 2023 provides a detailed description of research on NLRP3 targeted inhibitors and natural drug active ingredients, beginning with the inhibition of NLRP3 inflammasomes[34]. Conversely, research and development of pharmaceutical agents targeting the cGAS/STING signaling pathway offers novel avenues for the management of SICM.

**Table 1:** Potential treatments related to mtDNA

Name	Models	Effect	Reference
PHB1	Mouse (LPS)	1. Reduce mtDNA in mouse serum; 2. Reduce ROS generation, and improving mitochondrial biogenesis; 3. Promotes mPTP formation	[35]
BAM15	Mouse (CLP)		[36]
	Mouse (LPS)	Promote the conversion of M1 pro-inflammatory macrophages to M2 anti-inflammatory macrophages to exert an anti-inflammatory effect	[37]
TREM2^hi^ resident macrophages	Mouse (CLP)	Actively removes damaged mitochondria and inhibits heart inflammation	[38]
Chicoric acid	RAW264.7 cells Mouse (LPS)	The regulation of macrophage metabolic reprogramming	[39]
Dracorhodin	Mouse (LPS)	Targeting the CMPK2/NLRP3 signaling pathway	[40]
Ergolide		1. Covalently binds NLRP3; 2. Inhibit pyroptosis	[41]
Tangeretin		regulating PLK1/AMPK/DRP1 signaling axis, inhibiting ROS-mediated NLRP3 inflammasome activation	[42]
Echinatin		Targeting HSP90 inhibits NLRP3 inflammasome	[43]
Alda-1 (ALDH2 activator)		Reduces inflammation and apoptosis through the cGAS/STING pathway	[44]
Polysaccharides	Mouse (CLP)	Inhibiting the cGAS-STING signaling pathway	[45]

Mouse (LPS): a mouse model of sepsis induced by intraperitoneal injection of lipopolysaccharide; Mouse (CLP): mouse model of sepsis induced by cecum ligation; Cells (LPS): induction of septic cell models by lipopolysaccharide treatment

In addition, clinical studies have shown that plasma mtDNA levels correlate with mortality and prognosis in patients with sepsis[46]. A large number of mtDNA point mutations have been detected in the plasma of patients with septic shock, more than half of which are associated with ETC, ATP production and superoxide metabolism[47]; a novel sepsis diagnostic model for plasma mtDNA is being established, which is expected to be an innovative tool for the early diagnosis of sepsis[48].

### Mitochondrial calcium levels

Intracellular Ca^2+^ in cardiomyocytes is a widely distributed second messenger for a variety of physiological functions and is involved in the regulation of gene expression, cellular metabolism, and physiological processes such as cardiac contraction. Ca^2+^ level overload has been observed in numerous animal models of sepsis, where high levels of Ca^2+^ level in mitochondria destroy oxidative phosphorylation processes, leading to impaired energy supply to cardiomyocytes[49]. Clinical retrospective studies have shown that serum calcium levels in patients with sepsis correlate with mortality, and mRNA expression levels related to both mitochondrial energy production and ECC are decreased by more than 40%[50, 51], emphasizing the importance of monitoring serum calcium levels in patients. As a result, some progress has been made in the study of potential drugs for SICM along the lines of maintaining cellular Ca^2+^ homeostasis (Table 2).

**Table 2:** Ca^2+^ homeostasis-associated regulatory targets

Target	Name	Models	Effect	References
VDAC1	TMBIM6	Mouse (LPS)	1. Prevent VDAC1 multimerization 2. Improve mitochondrial quality control	[52]
	Balasubramide derivative		Tissue-specific anti-inflammatory activity	[53]
	SAMHD1	RAW264.7 cells; Mouse (LPS)	Maintain macrophage mitochondrial function	[54]
	Puerarin (TCM)	MVECs cells; Mouse (LPS)	1.Targeting the PGAM5-VDAC1 axis 2. Novel GAS6 Receptor Agonist	[55]
SERCA2a	Pyruvate kinase M2 (PKM2)	Mouse (LPS)	Maintaining calcium homeostasis	[56]
CaMKII	RCAN1		Activation of CaMKII exacerbates mitochondrial injury and SICM	[57]
	AC3-I	Mouse (CLP)	As a CaMKII inhibitory peptide	[58]
Calcium-binding protein	S100a8/a9		Activating ERK1/2-Drp1-mediated mitochondrial fission and respiratory dysfunction	[59]
IP3R2	Xestospongin C (XeC)	Rat (LPS), NVECs (LPS);	Activation of NLRP3/Caspase-1/GSDMD pathway	[60]

VDAC1; SERCA2a, sarcoplasmic/endoplasmic reticulum calcium ATPase 2a; CaMKII, calcium/calmodulin-dependent protein kinase II; IP3R2,the inositol 1,4,5-trisphosphate receptor type 2; NVECs, primary neonatal rat cardiomyocytes

## Mitochondrial rescue: biogenesis and autophagy

Mitochondrial biogenesis is a regenerative program that maintains mitochondrial populations by replacing aged and damaged mitochondria with new and healthy ones[61]. Mitochondrial autophagy represents a crucial aspect of the mitochondrial quality control system, encompassing the selective removal of superfluous and impaired mitochondria by lysosomes[62]. These two opposite processes reflect the highly dynamic nature of mitochondria, responding to changing cellular metabolic states and intracellular environments.

### Mitochondrial biogenesis in SIMD

ROS accumulation, NO accumulation, and ATP depletion are all activation signals for mitochondrial biogenesis, which are regulated by mtDNA and nuclear genes (nDNA) and multiple transcription factors. Cellular energy metabolism and mitochondrial biogenesis are closely linked through the SIRTs-PCG1α-Nrf1/2-TFAM pathway. Among the sirtuins family, SIRT1 is a nuclear protein that links the redox balance of cells to catabolism, regulates a variety of nuclear-encoded mitochondrial proteins and mitochondrial transcription factors (TFAM), and activates mitochondrial biogenesis[63]; SIRT3 is located in the mitochondria and can target glutamate dehydrogenase (GDH) and lactate dehydrogenase (LCAD) to improve activity, participate in mitochondrial glycolipid metabolism and mitochondrial biogenesis. In sepsis, SIRT1 affects the expression of mitochondrial SIRT3 through the pathway of the NF-κB transcription factor family member RELB[64]. Studies have shown that many natural drug active ingredients promote mitochondrial biogenesis through this pathway, improve mitochondrial quality, and improve septic-induced cardiac dysfunction (Table 3).

**Table 3:** Natural ingredients that improve mitochondrial biogenesis

Name	Models	Effect	References
Curcumin	HL-1 cells(LPS); mouse(LPS, ip)	Promotes mitochondrial biogenesis and inhibits mitochondrial fragmentation by activating SIRT1	[65]
ACE2 activator	Mouse(CLP)	1. Promoting MasR-Sirt1-mediated mitochondrial biogenesis 2. Inhibiting M1 macrophage via NF-κB/STAT1 signals	[66, 67]
Rosmarinic acid	Mouse(LPS, ip)	Activating Sirt1/PGC-1α pathway	[68]
Malvidin	Mouse(LPS, ip)	Promotes PGC-1α/Nrf2 signaling pathway	[69]
Songorine	Mouse(LPS, ip)	1. Activated Nrf2/ARE and NRF1 signaling cascades, 2.Wnt/β-catenin signaling pathway	[70] [71]
Myricanol	Mouse(LPS, ip)	SIRT1 activator, targeting inflammatory signal pathways and oxidative stress to suppress excessive inflammatory responses	[72]

### Mitochondrial autophagy

Mitophagy is essential for stabilizing the energy metabolism of cardiomyocytes and maintaining heart function in SICM[62]. A large amount of literature has reported the adaptive and maladaptive autophagy that occurs in the septic heart, that is, autophagy is increased in mild or early sepsis, and downregulated in severe or late sepsis. Therefore, some researchers have suggested using LC3II and p62 as biomarkers to monitor the autophagy flux in septic patients, which can help to intervene in autophagy at the appropriate time point[63]. Some research teams have demonstrated in mouse models that plant extracts (such as apigenin and phlorizin) and the traditional Chinese medicine compound preparation, Po-Ge-Jiu-Xin decoction (PGJXD), can improve septic myocardial injury by inducing autophagy[73, 74]. Two reviews provide detailed information on the current state of pharmacological research on the regulation of mitochondrial autophagy and explore the possibility of clinical translation[75, 76]. The University of Macau team has developed methodological standards for autophagy regulators, which will promote safer and more standardized research and development of mitochondrial autophagy regulators with drug translation potential[77]. This indicates that small molecule compounds targeting mitochondrial autophagy are becoming a trend in the research and development of new drugs for sepsis.

## Exploring potential treatment options

Although the mortality rate of sepsis is decreasing at a rate of 0.42% to 3.3% per year, this is closely related to the local level of medical care, especially for low- and middle-income countries, where the mortality rate of neonatal sepsis patients is as high as 88%. It is still an urgent global epidemiological problem that needs to be solved[78]. SIMD is the most common and serious complication. Due to its complex pathophysiological mechanism, there is still huge room for exploration in the clinical diagnosis, treatment and new drug research and development of sepsis.

### Regulation of overall cardiac energy metabolism

Abnormal myocardial mitochondrial energy metabolism has been reported in various cardiovascular diseases, such as myocardial hypertrophy, acute myocardial infarction, and chronic heart failure. The peroxisome proliferator-activated receptor (PPAR) family is a highly sought-after target for the development of drugs to treat metabolic syndrome and cardiovascular disease. Researchers have successfully developed a large number of PPARα agonists (fibrates) and PPARγ agonists (thiazolidinediones) for the clinical treatment of hyperlipidemia and type 2 diabetes. At the same time, animal experiments have confirmed that this pathway can improve the mitochondrial function of the heart in SICM mice and increase the survival rate of mice[79, 80]. Currently, levosimendan, milrinone, and norepinephrine are undergoing phase 4 clinical trials and are the most promising clinical drugs for SIMD.

### Lactic acid: worth reconsidering

In the SIMD, myocardial cells use lactate as their main energy substrate. The lactate shuttle theory suggests that lactate can be used as an energy substrate for exchange between tissues, organs or cells, and that the body unifies carbon sources in this way. This demonstrates the adaptive protection of the body under stress, providing a new understanding of the hyperlactatemia exhibited by most patients with sepsis upon admission to the hospital[81, 82]. This shows that traditional concepts are changing: the presence of lactic acid maintains the basic energy supply of the heart, and the level of lactic acid clearance in the later stages of sepsis may indicate the patient's ability to switch energy metabolism modes: that is, whether they can switch from aerobic glycolysis to oxidative phosphorylation, which is closely related to the patient's prognosis. Therefore, future clinical treatment should fully consider the important role of lactate as the main energy substrate in patients with sepsis.

### Non-coding RNA and nucleic acid drugs

With the development of molecular technology, non-coding RNAs (ncRNAs) will inject new vitality into the clinical diagnosis of SIMD and the research and development of nucleic acid drugs. lncRNAs and microRNAs are often associated with mitochondrial dysfunction and inflammation regulation in SICM (Table 4), and can be used as direct regulatory targets or novel biomarkers. Circular RNAs (circRNAs) may be involved in mitochondrial autophagy and biogenesis by acting as miRNA sponges (circ_0003907, circ_27393, etc.) [83, 84].

**Table 4:** Role of lncRNAs and microRNAs in SICM

Classify	Name	Models/samples	Effect	References
LncRNA	MCM3AP-AS1	HL-1 cells; Mouse (CLP); Clinical plasma samples(30)	Targeting the miR-501-3p/CADM1/STAT3 axis	[85]
		Clinical plasma samples(122)	1. As a new biomarker 2. Targeting the miR-28-5p/CASP2 Axis Regulates Inflammation and Apoptosis	[86]
	TTN-AS1	H9C2 cells; Rat (LPS)	Targeting the miR-29a/E2F2 Axis	[87]
	AABR07066529.3		Inhibiting MyD88 signaling pathway	[88]
	MALAT1	Rat (LPS)	Regulates USP22 expression through EZH2/ H3K27me3 axis	[89]
		Mouse (LPS)	1. Regulation of miR-146a-mediated mitochondrial autophagy 2. TLR4/NF-kB/MAPK signaling pathway	[90]
	MIAT	Clinical plasma samples:62; Rat (LPS)	Targeting the NF-κB axis	[91]
	GAS5、KCNQ1OT1	Clinical serum samples and PBMC	As a potential biomarker of sepsis severity and mortality risk	[92]
microRNA	miR-340-5p	Mouse (LPS)	Alleviates oxidative stress injury by targeting MyD88	[93]
	MiR-361-5p	Mouse (LPS)	Targeting Lgr4 to inhibit the Wnt axis	[94]
	MiR-702-3p	H9C2 cells (LPS);	Targeting NOD1	[95]
	MiR-539-5p		Targeting IRAK3	[96]
	MiR-31-5p		Targeting BAP1 to inhibit SLC7A11 deubiquitination	[97]

In addition to the regulation of key proteins, epigenetic modification site regulation is an emerging regulatory method. Among them, the role of histone modification and N6-methyladenosine (m6A) modification in energy metabolism and immune response in SIMD is being gradually revealed. It has been demonstrated using a SICM rat model that ECMO can alleviate the progression of septic cardiomyopathy by increasing m6A modification, which not only supplements the pathophysiological mechanism of SIMD, but also expands the scope of pharmacological research on SIMD[98, 99].

### Old medicine new use

Metformin is a first-line treatment for type 2 diabetes. It is characterized by high safety and low cost. In recent years, researchers have continued to explore its mechanism of action in an attempt to develop indications other than diabetes[100]. Recently, researchers used bioinformatics and network pharmacology databases to predict mitochondrial-related targets of sepsis, and demonstrated that metformin can restore mitochondrial function and improve cardiac function in SICM cell models and mouse models. In addition, it can also improve the metabolic status of macrophages and endothelial cells and inhibit excessive inflammatory responses[101]. A clinical retrospective study showed that metformin is effective in treating organ dysfunction caused by mitochondrial dysfunction in sepsis[102]. Meanwhile, three clinical studies are underway to evaluate the effectiveness and safety of metformin in patients with sepsis (NCT05979038, NCT06181422, NCT05900284).

Melatonin is an amine hormone produced by the pineal gland. It is mainly used to adjust circadian rhythms and treat insomnia, but recent studies have found that it has a variety of biological effects such as anti-inflammatory, antibacterial, antioxidant and cardioprotective effects. Based on this, exogenous melatonin has become an emerging drug for the treatment of sepsis. Experiments on mice and rats with sepsis have shown that melatonin can effectively reduce oxidative stress levels, inflammation and apoptosis in heart tissue[103]. As research has progressed, the protective mechanisms of melatonin on mitochondria have gradually been revealed, including promoting mitochondrial autophagy and biogenesis, enhancing the activity of the mitochondrial ETC complex, and protecting against damage caused by iNOS[104, 105]. Studies using ultrasound-targeted microbubble destruction (UTMD) technology have significantly enhanced the protective effect of melatonin on the heart function of septic rats at safe doses, and have demonstrated the biosafety of cardiac targeted delivery[106]. In addition, two clinical trials have shown that the use of melatonin can significantly improve the SOFA score in patients with sepsis and effectively reduce 28-day mortality when used in combination with high-dose VC. Furthermore, there are no side effects associated with intravenous administration[107, 108]. These experiments demonstrate the potential of melatonin in the treatment of sepsis.

### Mitochondrial input therapy

Autologous mitochondrial input therapy is a new alternative therapy, which infuses exogenous functional mitochondria into the lesion site through various routes to increase the number of mitochondria and improve mitochondrial function. It has been shown to effectively improve myocardial energy metabolism disorders in animal models of diabetic cardiomyopathy (DCM), myocardial infarction, and heart failure, enhance myocardial contractility, and reduce the size of the infarct area, which provides new ideas for the treatment of SIMD[109, 110].

## Conclusion

Metabolic disorders and energy failure in cardiomyocytes are typical features of the heart in SIMD patients, and mitochondrial damage and dysfunction are the main causes of this clinical manifestation. On the one hand, cardiomyocytes attempt to adaptively adjust to cope with the energy shortage; however, this adjustment, to some extent, causes mitochondrial respiratory dysfunction, oxidative stress, and calcium homeostasis imbalance, triggering mitochondrial DAMPs and MAMPs, which cause inflammation and cell death. On the other hand, the efficiency and coordination of mitochondrial autophagy and biogenesis are effective ways to repair and maintain mitochondrial function.

Therefore, we need to understand more about the mechanism of bioenergetic metabolism, explore effective biological targets, and aim to improve mitochondrial dysfunction and restore myocardial energy supply, with the aim of helping doctors identify different subtypes and stages of sepsis and develop personalized treatment strategies. In future research, controlling cardiac energy failure may be an effective treatment for SIMD.

## Literature search

Literature searches were conducted using PubMed and major journals of clinical and basic medicine. The search was set to the last 5 years, with no restrictions on the type of article. PubMed search keywords were: sepsis, mitochondria, sepsis cardiomyopathy, energy, metabolism, oxidative stress, mitochondrial biogenesis, etc. Combinations were screened, and related keywords were used to identify literature.

## Data Availability

The datasets used and/or analyzed are available from the corresponding author on reasonable request.
